# Genome-wide association studies on periodontitis: A systematic review

**DOI:** 10.1371/journal.pone.0306983

**Published:** 2024-09-06

**Authors:** Chenyi Gao, Mark Iles, Harriet Larvin, David Timothy Bishop, David Bunce, Mark Ide, Fanyiwen Sun, Susan Pavitt, Jianhua Wu, Jing Kang

**Affiliations:** 1 School of Dentistry, University of Leeds, Leeds, United Kingdom; 2 School of Medicine, University of Leeds, Leeds, United Kingdom; 3 Wolfson Institute of Population Health, Queen Mary, University of London, London, United Kingdom; 4 Leeds Institute of Medical Research, School of Medicine, University of Leeds, Leeds, United Kingdom; 5 School of Psychology, University of Leeds, Leeds, United Kingdom; 6 Centre for Host Microbial Interactions, Faculty of Dentistry Oral and Craniofacial Sciences, King’s College London, London, United Kingdom; 7 Oral Clinical Research Unit, Faculty of Dentistry Oral and Craniofacial Sciences, King’s College London, London, United Kingdom; University of Catania: Universita degli Studi di Catania, ITALY

## Abstract

**Objectives:**

This study aims to systematically review the existing literature and critically appraise the evidence of genome-wide association studies (GWAS) on periodontitis. This study also aims to synthesise the findings of genetic risk variants of periodontitis from included GWAS.

**Methods:**

A systematic search was conducted on PubMed, GWAS Catalog, MEDLINE, GLOBAL HEALTH and EMBASE via Ovid for GWAS on periodontitis. Only studies exploring single-nucleotide polymorphisms(SNPs) associated with periodontitis were eligible for inclusion. The quality of the GWAS was assessed using the Q-genie tool. Information such as study population, ethnicity, genomic data source, phenotypic characteristics(definition of periodontitis), and GWAS methods(quality control, analysis stages) were extracted. SNPs that reached conventional or suggestive GWAS significance level(5e-8 or 5e-06) were extracted and synthesized.

**Results:**

A total of 15 good-quality GWAS on periodontitis were included (Q-genie scores ranged from 38–50). There were huge heterogeneities among studies. There were 11 identified risk SNPs (rs242016, rs242014, rs10491972, rs242002, rs2978951, rs2738058, rs4284742, rs729876, rs149133391, rs1537415, rs12461706) at conventional GWAS significant level (*p<5x10*^*-8*^), and 41 at suggestive level (*p<5x10*^*-6*^), but no common SNPs were found between studies. Three SNPs (rs4284742 [G], rs11084095 [A], rs12461706 [T]) from three large studies were from the same gene region–SIGLEC5.

**Conclusion:**

GWAS of periodontitis showed high heterogeneity of methodology used and provided limited SNPs statistics, making identifying reliable risk SNPs challenging. A clear guidance in dental research with requirement of expectation to make GWAS statistics available to other investigators are needed.

## Introduction

Periodontitis is a common disease affecting the tissues supporting and surrounding the teeth [1]. This disease is a major cause of tooth loss and is associated with a range of multiple long-term chronic conditions (e.g., diabetes [2, 3], cardiovascular diseases [4, 5], and cognitive impairments [6–9], which can negatively impact quality of life and increase the risk of mortality [10]. Because of the effect of periodontitis, periodontal treatment is critical to the patients and several recent periodontal treatment approaches has found more effectively reduce periodontitis [11] and also the inflammation mediator (C-reactive protein) that participated in both periodontitis and other systemic disease (i.e., cardiovascular disease) [12]. Despite of evolving periodontal treatments, how do we define the periodontitis is vitally important in either investigating the pathology but also in following treatment approaches. Although there have been consensus reports on universally accepted periodontitis definition such as for the 1999 classification of periodontal diseases [13–15]; and then later the 2018 AAP/EFP classification of periodontal and peri-implant diseases and conditions [16]), the actual definition of periodontitis employed in dental studies shows considerable heterogeneity.

Understanding of the pathology and aetiology of periodontitis is fundamental to improve the treatment approaches of periodontitis. Based on the current understanding, the pathology and aetiology of periodontitis is complex and multifactorial, including microorganism pathogens, environmental factors, lifestyle behaviours (such as nutrition, oral hygiene, and smoking) [2], epigenetic factors [17] and genetic factors [2, 18]. By focusing on the genetic impact on periodontitis, a recent systematic review on heritability of periodontitis suggested 7%-38% across different study designs (e.g., twin study, other family study, or genome-wide association study (GWAS)) [19]. GWAS comprehensively investigates the association between a trait or diseases and hundreds of thousands of genetic variants (most commonly single nucleotide polymorphisms, SNPs) across the genome [20]; the technique is considered agnostic in terms of not depending on any aspect of the disease biology. GWAS as a technique aims to identify SNPs statistically associated with the trait or disease of interest, so called genetic risk variants or loci. The statistical technique involves comparing the allele frequency differences between cases (with the trait in question) and controls (persons without the trait of interest). The GWA approach has been applied to most common diseases since technology allowed the conduct of such studies in 2005 [21]. Since then, this GWA technique has been applied to oral diseases including periodontitis, contributing to finding more SNPs/genes associated with periodontitis, complementing genetic studies based on biological mechanisms proposed to influence the likelihood of periodontitis.

Current GWAS of periodontitis face many challenges, most notably limited sample size, population stratification, variation in methodologies applied, and use of non-consensus definitions of periodontitis, that complicate interpretation of the consistency of the results [20]. Even though there are some reviews [17, 22, 23] on the genetic aspects of periodontitis, there have been limited attempts to systematically evaluate GWAS studies of periodontitis. To date, there is only one systematic review on the heritability of periodontitis [19], and one descriptive review of periodontitis [23].

Since more GWAS on periodontitis have been published recently, a systematic evaluation and, ideally meta-analysis, of the available evidence would improve the understanding of the genetic mechanisms of periodontitis, address the current research gap, and contribute to better design and analysis of future GWAS. The aim of this systematic review on periodontitis is to: 1) critically appraise the evidence of GWAS of periodontitis. 2) Synthesise the findings and results by summarizing SNPs identified by high quality GWAS, and 3) meta-analyse appropriate studies/SNPs if summary statistics of GWAS were available.

## Materials and methods

The study was to systematically review the GWAS that explored the genetic risk factors associated with periodontitis in the general population. This study is registered at the PROSPERO platform (ID: CRD42023456388). The main amendment in this manuscript compares to the protocol is that the meta-analysis was not performed due to appropriate data’s unavailability.

### Search strategy

This systematic review was conducted according to the Preferred Reporting Items for Systematic reviews and Meta-Analyses (PRISMA) statement 2020 [24], as well as the guideline for performing systematic review for gene association studies [25]. The search was conducted on PubMed, the GWAS catalog, the ScienceDirect, the Embase, Global Health, Medline via Ovid to ensure the broad coverage of GWAS. The details search strategies in each database were summarised in S1-S4 Tables in S1 File. The search term including periodontitis (e.g., periodontal disease, periodontitis, periodon*) and GWAS terms (e.g., GWA, GWAS, genome wide association, whole genome association, WGA, WGAS). The “*” sign is to indicate any words start with “periodon”.

PEO: The Population of this study is human participants. The Exposure of this study is the genetic variants (SNPs). The Outcome of this study is ‘periodontitis’.

The search was restricted to the period 2005–2023 to limit the irrelevant genetic study, since GWAS study were not conducted prior to 2006. The search was completed on 31 Aug 2023.

### Study selection

Strict eligibility criteria were developed by the four authors through detailed discussion to ensure relevant study inclusion:

1. Study design is a genome-wide association study on human population including all ethnicities.
2. Study disease/primary outcome/disease phenotype is of any form of periodontitis, including clinically diagnosed periodontitis with any diagnosis criteria, or self-reported. We do not specify the heritability, power, allele frequency at the stage of the search but will synthesize such information at the data extraction.

Exclusion criteria are as below:

1. The study is a genetic/candidate gene study but not a whole genome wide scan for risk/protective SNPs of periodontitis.
2. The study used oral pathogen or gingivitis as a proxy for periodontitis. Oral pathogens and gingivitis were not considered as periodontitis as they did not directly imply the presence of periodontitis.

Search results were downloaded and imported to Covidence (link: https://get.covidence.org/literature-review?campaignid=18165361407&adgroupid=138405766537&gclid=EAIaIQobChMIwfHnroSs_AIVTLDtCh10zAAEEAAYASAAEgJP_fD_BwE) for study screening and eligibility assessment. First, duplication screening and removal is automatically done by COVIDENCE. Then, a three stage of eligibility assessment performed, and this consists of: initial abstract screening, full text screening and conflict resolve. Two authors (CG, JK) processed the abstract screening to narrow down the number of studies to be include in full text screening and eligibility assessment where all study might potentially include a GWA analysis on periodontitis is marked as “include” or “Maybe”. All “include” and “Maybe” marked study were screened for its full text to confirm its eligibility where GWAS on periodontitis or study include GWA analysis on periodontitis were marked as “include” only. Papers where the two authors had differing opinions regarding inclusion or exclusion were then screened by two more authors (HL, FS) and discussed by all four authors for the final decision. The eligibility of papers was confirmed before data extraction and quality assessment. A data extraction form was developed and based on the study [26]. The information of SNP location and chromosome, and the nearest gene was recorded from dbSNP (https://www.ncbi.nlm.nih.gov/snp/) based on the Genome Reference Consortium (GRC) released "build 37" of the human genome (GRCh37) version to keep the consistency of the SNP location.

### Data extraction

The steps for identifying genome-wide associated significant SNPs vary across studies, and one or two or three steps were applied among the included GWAS: 1) Conducting statistical test for association on one dataset only—discovery stage; 2) Conducting meta-analysis on (an)other dataset(s)—meta-analysis stage, optional; or 3) Further seeking an independent replication / validation using other dataset(s), optional. Sometimes step 2 and 3 can swop [20].

In this systematic review, no study was excluded by including replication or meta-analysis stage or not, but SNPs with *p<5x10*^*-6*^ were extracted from the final stage of each identified study. SNPs with conventional GWAS significance (*p<5x10*^*-8*^) were also highlighted. For example, some studies contained discovery, validation/replication, and meta-analysis stage, so we extracted only the SNPs that were significant at the meta-analysis stage. If a study performed GWAS association analysis at discovery stage only, significant SNPs from discovery stage were extracted. The essential statistics and information were extracted on Excel sheets, including SNP ID, Chromosome and position, Odds Ratio or Coefficient Beta, Standard error or confidence interval, Nearest Gene, risk allele, ethnicity, sample size, quality control procedure, periodontitis definition etc. However, due to the unavailability of full list summary statistics from almost all studies (except Shungin et al. 2019), the further meta-analysis cannot be performed. Additional information (i.e, participants number, age, ethnicity, participants inclusion/exclusion criteria, data resource, type of periodontitis, clinical phenotype: measurements & definitions, study design: GWAS stage included, quality control during analysis, GWAS significant threshold, statistical model in GWAS) on methodology were also extracted.

### Quality assessment

The Q-Genie tool, a validated tool developed by McMaster University for rating the quality of genetic association studies [27, 28], for GWAS quality assessment was used. This tool includes 11 assessment areas including rationale, outcome classification, comparison groups, exposure, source of bias, power analysis, statistical methods used, test of assumptions and inferences, and conclusion, with each area scored max 5, making the total highest score 55. All papers were assessed by two authors in parallel, and any deviation between individual ratings was discussed and validated by a third author.

In addition to the Q-Genie tool, we also checked the quality control procedure in each included paper following the guidance of GWAS quality control (i.e., call rate, HWE, MAF, relatedness, population stratification, heterozygosity rate, sex mismatch) [20, 29, 30]. We have also extracted the genomic inflation, reference alleles and allele frequency from the 1000 Genomes allele frequency table from dbSNP for checking the inflation reported and comparing the reported alleles and allele frequency [31]. Additional information such as covariates controlled in the association analysis model and imputation quality check were also extracted from each study.

## Results

### Study selection

**Fig 1:**
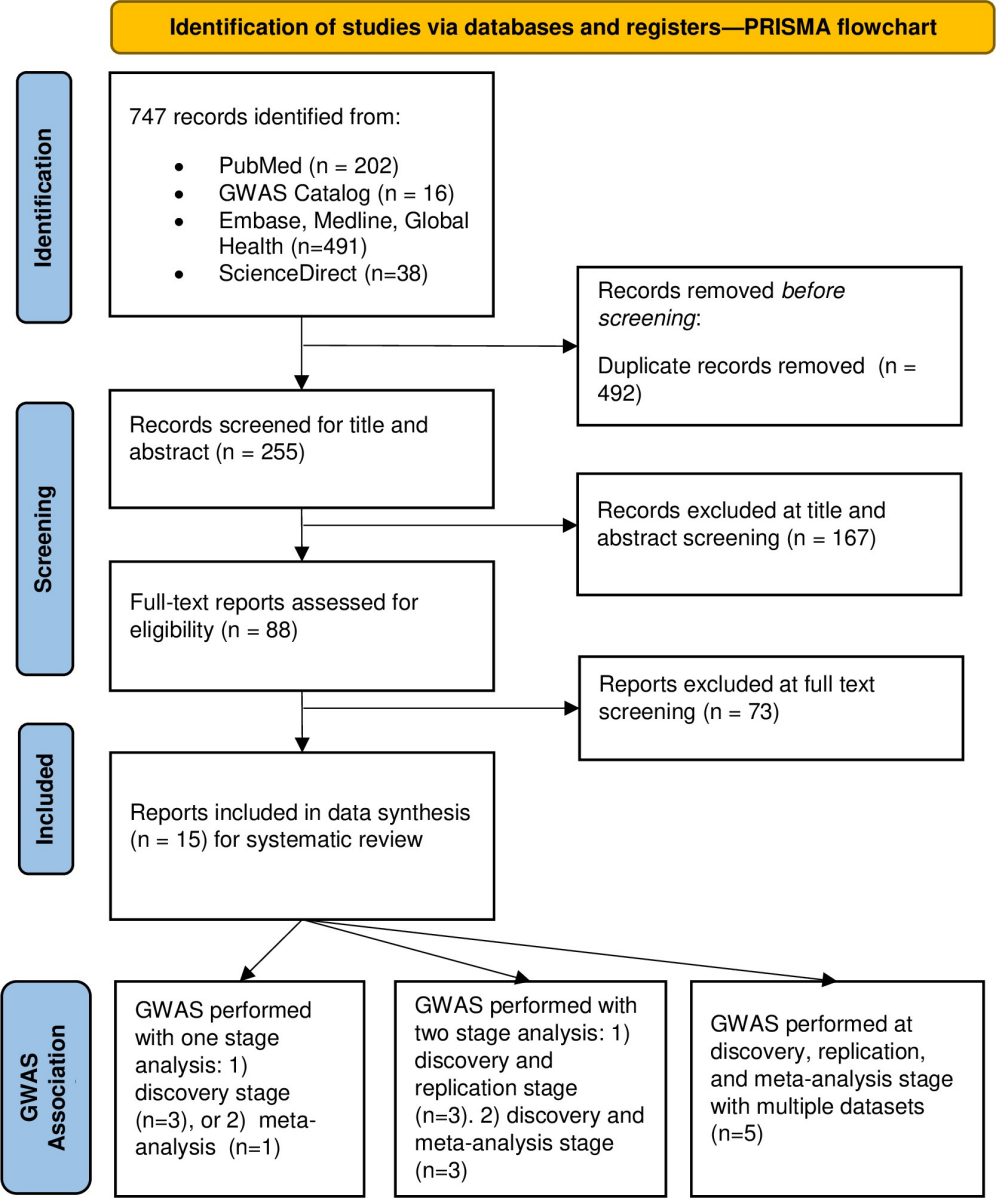
The PRISMA flow chart of the study inclusion process.

After the systematic search in GWAS catalog and PubMed, there were 16 papers from GWAS catalog, 202 papers from PubMed, 491 papers from EMBASE, Global Health and Medline Via Ovid, and 38 papers from ScienceDirect retrieved. After removal of duplicate studies and screening the abstract, 88 papers’ full text were assessed for eligibility. Of these, 15 papers were deemed eligible [32–46]. All included studies were published between 2010 and 2023. Fig 1 displays the PRISMA flow chart, and PRISMA 2020 checklist is provided as supplement (S7 Table in S1 File and S8 Table in S1 Checklist).

### Quality assessment

The Q-Genie tool was applied to assess the quality of each eligible study. Overall, the studies have satisfactory quality (scores ranged from 38–50, Table 1). However, the quality varies regarding the classification of the outcome, description of comparison groups, whether the study is adequately powered, statistical methods, description of the test and inferences (scores varied from 2 to 5 in each area among included GWAS). It is worth noting that many studies have insufficient sample size, and only five studies [34, 37, 39, 44, 45] each had a total sample over 10,000 (Table 2).

**Table 1:** Quality assessment rating of all 15 included studies using Q-genie tool.

	Paper	Adequacy of the presented hypothesis and rationale	Classification of the outcome	Description of comparison groups	Technical classification of the exposure	Non-technical classification of the exposure	Disclosure and discussion of sources of bias	Study adequately powered	Description of planned analyses	Statistical methods	Description and test of all assumptions and inferences	Conclusions drawn by the authors were supported by the results and appropriate methods	Overall
1	Bevilacqua et al. (2017)	4	5	3	5	3	4	1	4	4	1	4	38
2	de Coo et al. (2021)	5	5	3	6	5	4	2	4	4	4	4	46
3	Divaris et al. (2013)	5	5	3	6	6	4	3	4	4	5	5	50
4	Feng et al. (2014)	4	4	3	4	3	2	2	4	4	4	3	37
5	Hong et al. (2015)	4	5	3	5	4	3	2	4	4	4	5	43
6	Munz et al. (2017)	4	4	4	5	4	2	4	5	5	4	5	46
7	Munz et al. (2019)	4	4	4	5	4	2	5	5	5	4	5	47
8	Sanders et al. (2017)	4	4	2	4	4	3	5	4	5	4	5	44
9	Schaefer et al. (2010)	5	3	4	4	3	2	2	4	4	4	4	39
10	Shaffer et al. (2014)	4	5	2	4	4	3	2	4	4	4	5	41
11	Shimizu et al. (2015)	5	3	3	4	4	5	5	5	5	4	5	48
12	Shungin et al. (2019)	5	2	3	5	4	5	5	4	5	4	5	47
13	Tegelberg et al. (2021)	2	2	2	3	3	4	2	4	2	4	5	33
14	Teumer et al. (2013)	4	4	2	3	5	4	2	3	4	4	4	39
15	Petty et al., (2023)	4	5	3	5	3	4	2	4	4	4	4	42

**Table 2:** Summary of all the 15 included studies.

Study	participants number (in analysis)		age	ethnicity	data resource	type of periodontitis	definition of periodontitis	GWAS stage included
	case	control						
Bevilacqua et al., (2018)	442	160	18–89 years old	Italian (isolated sample)	‘Friuli Venezia Giulia Genetic Park’project	CP	the criteria of the AAP (Armitage, 1999)	discovery stage
de Coo et al., (2021)	441	1141	14–35 years old	Spanish	The PerioGEN study	AgP	the classification of periodontal diseases [60, 61]	discovery stage
Divaris et al., (2013)	3251	1909	53–79 years old	European American	(1) the Atherosclerosis Risk in Communities (ARIC) cohort. (2) the Health, Aging and Body Composition (Health ABC) Study	CP	the CDC-AAP consensus	(1) discovery stage. (2) replication stage. (3) meta-analysis stage
Feng et al., (2014)	712	1973	25–89 years old	White, Black, other	(1) the Dental Registry and DNA Repository (DRDR) of the University of Pittsburgh School of Dental Medicine. (2)two independent population-based cohorts from Brazil (Porto Alegre & Rio de Janeiro)	CP	30% or more teeth with sites of clinical AL of five millimeters or more	(1) discovery stage. (2) replication stage. (3) meta-analysis stage
Hong et al., (2015)	414	263	44–88 years olds	Korean	the Korean Genome and Epidemiologic Study (KoGES) cohort	periodontitis	the CDC-AAP criteria (Eke et al. 2012).	Discovery stage: (1) GWAS overall population. (2) validate the SNPs in the subpopulation: moderate case, severe cases vs. healthy control.
Munz et al., (2017)	2067	8819	mean age 32–74 years	Dutch, German, Turkish	(1) the biobank Popgen. (2) the Competence Network "FoCus—Food Chain Plus" & the Dortmunder Gesundheitsstudie—DOGS. (3) recruited Turkish participants described in other study (Shaefer et al., 2015; Renauer et al., 2015)	AgP & CP	(1) CP: the number of proximal sites with AL> = 4 mm. (2) AP: ≥2 teeth with 30% alveolar bone loss under the age of 35 and no diabetes (Shaefer et al., 2015)	(1) Two GWAS performed in discovery stage. (2) replication stage. (3)two meta-analysis performed (1. discovery sample meta-analysis, all sample meta-analysis)
Munz et al., (2019)	5095	9908		Dutch, German, European American	(1) the biobank Popgen. (2) the Competence Network “FoCus–Food Chain Plus”, the Dortmunder Gesundheitsstudie–DOGS and the Heinz Nixdorf Recall Studies. (3) the ACTA (Academisch Centrum Tandheelkunde Amsterdam) and Rotterdam and Wageningen by the B-Proof Study. (4)the Atherosclerosis Risk in Communities (ARIC) Study. (5) a meta-analysis of SHIP and SHIPTREND cohorts	AgP & CP	(1)Dutch AgP: were ≥2 affected teeth with ≥ 30% bone loss in patients <36 years of age. (2) European American CP: the CDC-AAP (Page & Eke., 2007). (3) German CP: subjects within the first and the third tertile of proportion of proximal sites with AL ≥ 4 mm were contrasted after stratification by sex and 10-year age groups Age-specific tertiles were defined to include severely diseased cases within each age stratum.	meta-analysis stage
Sanders et al., (2017)	(1) discovery stage: 10935. (2) replication stage: 4402		18–76 years old	Hispanic/Latino, European American, African American	(1) The Hispanic Community Health Study / Study of Latinos (HCHS/SOL). (2)The ARIC cohort (1987 to present)	CP	4 case definitions from the CDC-AAP	(1) discovery stage. (2) replication stage.
Schaefer et al., (2010)	438	1320	< = 35 years old	Dutch, German	(1) the cases in discovery stage weas recruited from Germany from 2003–2008. (2) The control population in the discovery stage was recruited from the popgen biobank. (3) the replication sample was recruited from Netherlands between 2003–2006	AgP	(1) localized AgP: > = 50% bone loss at 2–6 teeth. (2) generalized AgP was characterized by > = 50% bone loss at > = 7 teeth. (Shaefer et al., 2009)	(1) two GWAS performed at discovery stage (GWAS1 & GWAS2). (2) replication stage. (3) meta-analysis stage
Shaffer et al., (2014)	(1) PD1: 176 (2) PD2: 93	(1) PD1: 497 (2) PD2: 529	18–49 years old	non- Hispanic Caucasian	the Centre for Oral Health Research in Appalachia	CP	Two or more sextants with probing depths of > = 5.5mm: (1) PD1 assumed edentulous sextants had not been affected by CP; (2) PD2 assumed the missing teeth in edentulous sextants had been affected by CP.	two GWAs performed at discovery stage (i.e., PD1, PD2)
Shimizu et al., (2015)	2760	15158	17–98 years old	Japanese	the Biobank Japan and participants recruited from Health Sciences University of Hokkaido and Tokyo Medical and Dental University, the Rotary Club of Osaka-Midosuji District, the PharmaSNP Consortium.	Periodontitis	criteria of the AAP (Armitage 2004; Armitage and Cullinan 2004, 2010; Page and Eke 2007)	(1) discovery stage. (2) validation stage. (3) meta-analysis stage
Shungin et al., (2019)	36332	470262	UK Biobank 40–69 years old	Mixed ethnicity	GLIDE & UK biobank	Periodontitis and loose tooth	(1) UK biobank: self-reported oral health questionnaire. (2) GLIDE: ARIC, SHIP, SHIP-Trend and HCHS/SOL (criteria published by the CDC-AAP (Page & Eke, 2007)); COHRA1 (> = 2 sextants had probing depth of > = 5.5 mm, or ever having ‘gum surgery’); TwinGene and MDC (at least two tooth surfaces had probing depth of > = 5 mm, or at least four tooth surfaces had probing depth of > = 4 mm); BBJ (clinical diagnosis by physicians at participating hospitals); TMDUAGP (the 1999 international workshop for a classification of periodontal disease and conditions (Armitage, 1999));WGHS (self-reported periodontitis or not)	(1) discovery stage. (2) meta-analysis stage
Tegelberg et al., (2021)	3245		30–65 years old34	Finish	(1) the national Health 2000 Survey. (2) the Northern Finland Birth Cohort 1966 Study	periodontal pocketing	The number of teeth with≥4 mm deep periodontal pockets	(1) discovery stage (two GWAS performed) (2) meta-analysis stage
Teumer et al., (2013)	(1) SHIP: 3365 (44.8% controls). (2) SHIP-TREND: 667 (46.4% controls)		20–79 years old	German	(1) The Study of Health in Pomerania (SHIP). (2) SHIP-TREND	CP	four different periodontitis case definitions included: (1) mean proximal AL (mean PAL), (2) proportion of proximal sites with AL ≥ 4 mm, (3) the CDC-AAP case definition (Page & Eke 2007)	(1) discovery stage: four GWAS were performed for four phenotype definitions. (2) meta-analysis stage: combined analysis of results from GWAS of PAL and Severe &moderate periodontitis
Petty et al., (2023)	333	546	>18 years old	Mixed ethnicity: African, European, East Asian, Hispanic/Latino, south Asian, Other	patients received treatment in at the UTHealth School of Dentistry or at the University of Pittsburgh School of Dental Medicine	AP	An AP lesion was characterized radiographically as a rarefaction lesion with the disappearance of the periodontal ligament space and discontinuity of the lamina dura.	(1) discovery stage. (2) validation stage

AL, attachment loss; AP, Apical periodontitis; AgP, Aggressive periodontitis; CP, Chronic periodontitis; CDC-AAP, the Centers of Disease Control American; Academy of Periodontology; AAP, American Academy of Periodontology. The full table with more detailed information summary of the included studies is included in the supplements (Stable 1).

For quality control procedure performed, all included studies have had or reported quality control steps taken but varied from 3 steps reported to 11 steps reported (Table 3). There were 5 studies did not report the genomic inflation score or linkage disequilibrium score regression intercept for inflation check. The full details of the quality control step taken in each study can be viewed in S5 Table in S2 File. Most included SNPs have similar effect allele or minor allele frequency reported but few SNPs with mismatch allele as the 1000 Genomes project were also noted (e.g., rs4242220, rs12969041, rs2027756) and effect allele or minor allele from one study was not reported (Table 4).

**Table 3:** Summary of quality control steps included in the 15 studies.

Study	Sample Call rate	SNP call rate	HWE	MAF	Relatedness	population stratification	Heterozygosity rate	Sex Mismatch	Imputation quality check	adjusted covariates in the statistics model	inflation check (LDSC intercept/GC lambda)
Bevilacqua et al., (2018)					√	√				sex, age, smoking	
de Coo et al., (2021)	√	√	√		√	√				gender, age and the first PC	
Divaris et al., (2013)	√	√	√	√	√	√		√	√	GWAS use ARIC data at discovery stage adjusted for age, sex, examination centre and first 10PCs)	GWAS in ARIC cohort (Discovery stage):λ = 1.019 for moderate and 1.024 for severe CP.
Feng et al., (2014)		√	√	√						1) discovery stage: age, sex, diabetes status, and smoking status, first five PCs adjusted in the complete dataset for managing population structure.2) replication stage: age, sex, ethnicity, diabetes status, and smoking status. BMI additionally adjusted in the sample from Porto Alegre	
Hong et al., (2015)		√	√	√	√	√	√	√		age, sex, smoking, drinking, education and BMI.	
Munz et al., (2017)	√	√	√	√	√	√		√	√	sex, smoking status and 6 MDSCs (German only).	1)before adjustment: λ = 1.08 for German and λ = 1.01 for Netherland. 2) after adjusting 1–6 MDSC in German: λ = 1.04
Munz et al., (2019)			√	√					√		the P-value correction method used for inflation management
Sanders et al., (2017)	√	√	√	√	√	√		√	√	1) discovery stage adjusted for the fixed effects of sex, age, field centre, cigarette use, sampling weights, genetic subgroup and 5 genetic principal components representing ancestry, and the random effects of census block group, household, and kinship. 2) replication stage adjusted for age, sex, smoking, and 10 ancestry principal components	λ = 0.990
Schaefer et al., (2010)	√	√	√	√							λ = 1.04 in stage 1 and 1.12 in stage 2.
Shaffer et al., (2014)			√	√	√	√				age	λ = 0.997 and 0.991 for PD1 and PD2, respectively.
Shimizu et al., (2015)		√	√	√	√	√					λ = 1.024
Shungin et al., (2019)	√	√	√	√	√	√	√	√	√	1) UK Biobank GWAS: Age, age squared, sex and genotyping array were as covariates in association testing. 2) GLIDE GWAS for the binary trait of periodontitis, age, age-squared and other study-specific covariates were instead included as covariates in association tests.	1) Combined: λ_GC_ = 1.09, h2 LDSR = 0.046, LDSR-i = 1.00 for periodontitis/loose teeth). 2) GLIDE: Association analysis results corrected by λ = 1.07
Tegelberg et al., (2021)	√	√	√	√						age, sex, smoking, plaque, and the first ten principal components	1) age, sex, and the first ten PCs adjusted: λ = 0.99 for NFBC66 and 1.01 for T2000 data. 2) age, sex, smoking, and the first ten PCs adjusted: λ = 0.98 for NFBC66 and 0.99 for T2000 data. 3) age, sex, smoking, plaque, and the first ten PCs: λ = 0.98 for NFBC66 and 0.99 for T2000 data.
Teumer et al., (2013)		√	√	√					√		The pvalues and SEs of both the individual cohort results and combined analysis results were corrected for genomic control if kGC was >1.
Petty et al., (2023)	√		√	√	√	√	√	√	√		

SD, Standard deviation; PCA, principal components analysis; HWE, Hardy–Weinberg equilibrium; LDSR, linkage disequilibrium score regression; LDSCR-I, LDSR intercept; GC, genomic control; IBS, pair-wise identity-by-state, MDSC, Multimentional scaling components; MDS, multimentional scaling; λ, GC lambda/genomic inflation factor; SE, standard error; AF, allele frequency. The full details of the quality control method can be viewed in the Stable 5

**Table 4:** The top signal significant SNPs at 5e-6 significant level of periodontal disease extracted from the 13 included papers.

Study	SNP	Chromosome: Position	OR (95% CI)	effect allele	EAF (or MAF)	other allele	p-value	Nearest Gene	1000Genomes alleles and allele frequency	Reference data ethnicity
Bevilacqua et al. (2017)	rs242016	12:3788260	3.7 (2.32–6.29)	A	0.21	G	1.50E-08	CRACR2A	G = 0.8469 A = 0.1531	Europe
	rs242014	12:3789135	3.7 (2.32–6.29)	T	0.2	C	1.60E-08	CRACR2A	C = 0.8469 T = 0.1531	Europe
	rs10491972	12:3789949	3.7 (2.32–6.29)	G	0.2	A	1.70E-08	CRACR2A	A = 0.8469 G = 0.1531	Europe
	rs242002	12:3807915	3.6 (2.28–6.13)	T	0.21	G	2.73E-08	CRACR2A	G = 0.8479 T = 0.1521	Europe
de Coo et al. (2021)	rs35709256	11:926122823	2.07 (1.55–2.77)	A	Cases 0.12 / Controls 0.07		9.48E-07	FAT3	G = 0.9056 A = 0.0944	Europe
	rs4807188	19:1975217	2.8 (1.83–4.27)	A	Cases 0.05/ Controls 0.02		1.81E-06	CSNK1G2	G = 0.9841 A = 0.0159	Europe
	rs2074872	17:10222061	2.12 (1.55–2.91)	A	Cases 0.41/ Controls 0.33		2.84E-06	MYH13, LOC107985004	G = 0.6054 A = 0.3946	Europe
	rs116611488	1:205005227	4.22 (2.31–7.72)	T	Cases 0.03/ Controls 0.009		2.90E-06	none	C = 0.9861 T = 0.0139	Europe
	rs4854545	2:69313773	4.34 (2.34–8.02)	G	Cases 0.03/ Controls 0.009		2.97E-06	ANTXR1	G = 0.0099 T = 0.9901	Europe
	rs78672540	8:5247045	2.32 (1.62–3.31)	C	Cases 0.08/ Controls 0.04		3.78E-06	**none**	T = 0.9334 C = 0.0666	Europe
	rs13439823	8:108440377	2.01 (1.49–2.7)	A	Cases 0.43/Controls 0.37		4.22E-06	ANGPT1	G = 0.6054 A = 0.3946	Europe
	rs11993287	8:144966243	0.58 (0.46–0.73)	A	Cases 0.31/ Controls 0.38		4.31E-06	none	G = 0.6750 A = 0.3250	Europe
Divaris et al. (2013)	rs2521634	7:24378040	1.49 (1.28–1.73)	G	Cases 0.80/ Controls 0.74	A	3.50E-07	LOC107986777	G = 0.830 A = 0.170	American
	rs7762544	6:41379315	1.4 (1.24–1.59)	G	Cases 0.21/ Controls 0.16	A	7.50E-08	none	G = 0.171 A = 0.829	American
	rs3826782	19:6887736	2.01 (1.52–2.65)	A	Cases 0.05/ Controls 0.04	G	8.20E-07	ADGRE1	G = 0.840 A = 0.160	American
Hong et al. (2015)	rs4242220	5:166744741	0.53 (0.41–0.69)	minor allele: C	0.24	A	2.84E-06	TENM2	T = 0.7381 G = 0.2619	East Asian
	rs12969041	18:13191184	2.86 (1.92–4.27)	minor allele: A	0.28	G	2.79E-07	none	C = 0.7431 T = 0.2569	East Asian
	rs2027756	18:13190107	2.86 (1.92–4.27)	minor allele: A	0.28	G	2.79E-07	none	C = 0.753 A = 0.000, T = 0.247	East Asian (1000 Genomes_3x)
Munz et al. (2017)	rs2978951	8:6823295	1.25 (1.16–1.35)	A	0.41	G	2.06E-08	none	A = 0.3966 G = 0.6034	Europe
	rs2738058	8:6821617	1.28 (1.18–1.38)	T	0.43	C	6.78E-10	none	T = 0.4175 C = 0.5825	Europe
	rs4284742	19:52131733	1.34 (1.21–1.48)	G	0.76	A	1.34E-08	SIGLEC5	A = 0.2584 G = 0.7416	Europe
	rs4970469	1:27312816	1.52 (1.29–1.81)	G	0.9	A	1.20E-06	none	G = 0.8598 A = 0.1402	Europe
	rs1122900	5:36689181	1.27 (1.16–1.4)	A	0.4	C	8.00E-07	none	A = 0.4414 C = 0.5586	Europe
	rs2070901	1:161185058	1.29 (1.16–1.44)	T	0.24	G	4.36E-06	FCER1G	G = 0.7306 T = 0.2694	Europe
Munz et al. (2019)	rs729876	16:13388778	1.23 (1.15–1.32)	T	1) German (Aggressive periodontitis): Cases 0.84/ Controls 0.80. 2) Netherlands: Cases 0.81/ Controls 0.79. 3) European American (severe): Cases 0.84 / Controls 0.80. 4) European American (moderate): Cases 0.82 /Controls 0.80.	C	1.21E-08	LOC107984137	T = 0.8990 C = 0.1010	Global
	rs11084095	19:52127030	1.17 (1.11–1.24)	A	1) German (Aggressive periodontitis): Cases 0.46/ Controls 0.41. 2) Netherlands: Cases 0.47/ Controls 0.42. 3) European American (severe): Cases 0.41 / Controls 0.39. 4)European American (moderate): Cases 0.42 /Controls 0.39. 5) German (Chronic Periodontitis): Cases 0.83/Controls 0.80	G	5.09E-08	SIGLEC5—AC018755.18	G = 0.7829 A = 0.2171	Global
	rs9982623	21:47691216	1.23 (1.13–1.33)	C	1) German (Aggressive periodontitis): Cases 0.88/ Controls 0.86. 2) Netherlands: Cases 0.88/ Controls 0.87. 3) European American (severe): Cases 0.89 / Controls 0.86. 4)European American (moderate): Cases 0.89 /Controls 0.86. 5) German (Chronic Periodontitis): Cases 0.86/Controls 0.86	T	8.65E-07	MCM3AP	C = 0.8660 T = 0.1340	Global
Sanders et al. (2017)	rs149133391	1:231716531	beta: -0.139 (-0.09 — -0.19)	T	MAF = 0.011	C	7.90E-09	TSNAX-DISC1	T = 0.9888 C = 0.0112	Global
	rs75715012	11:21649150	beta: 0.045 (0.03–0.06)	G	MAF = 0.089	A	1.10E-07	none	G = 0.9119 A = 0.0881	Global
	rs186066047	5:3875774	beta: 0.23 (0.14–0.31)	G	MAF = 0.003	A	1.70E-07	none	G = 0.9966 A = 0.0034	Global
	rs10456847	6:18955171	beta: -0.03(-0.04 — -0.02)	C	MAF = 0.330	G	2.60E-07	none	C = 0.6316 G = 0.3684	Global
	rs79308117	1:155479179	beta: 0.18 (0.11–0.25)	A	MAF = 0.005	C	2.80E-07	ASH1L	A = 0.9870 C = 0.0130	Global
Schaefer et al. (2010)	rs1537415	9:138529722	1.59 (1.36–1.86)	G	1) GWAS 1: MAF = Cases 0.5/ Controls 0.375. 2) GWAS 2: MAF = Cases 0.493/ Controls 0.371. 3) Validation: MAF = Cases 0.49/ Controls 0.396	C	5.51E-09	GLT6D1	G = 0.5845 C = 0.4155	Europe
Shaffer et al. (2014)	rs733048	4:13242797	2.4 ()	not found	1) PD 1: MAF = 0.22, 2) PD2: MAF = 0.22		1.00E-06	LOC105374494	G = 0.745 A = 0.255	American
	rs10457525	6:129872966	2.33 ()	not found	1) PD 1: MAF = 0.80		3.50E-06	LOC102723409	G = 0.744 T = 0.256	American
	rs7749983	6:129874355	2.39 ()	not found	1) PD 1: MAF = 0.19		2.40E-06	LOC102723409	T = 0.768 A = 0.232	American
Shimizu et al. (2015)	rs9446777	6:73581051	0.86 (0.80–0.93)	A	1)GWAS: MAF = Cases 0.12 /Controls 0.14, 2)replication: MAF = Cases0.13/ Controls 0.15	G	4.83E-06	KCNQ5	A = 0.8333 G = 0.1667	East Asian
	rs2392510	7:37746569	0.87 (0.82–0.92)	C	1)GWAS: MAF = Cases 0.37 /Controls 0.41, 2)replication: MAF = Cases0.37/ Controls 0.40	T	4.17E-06	GPR141	C = 0.4712 T = 0.5288	East Asian
Shungin et al. (2019)	rs12461706	19:52121235	1.05 ()	T	0.4		3.90E-09	SIGLEC5	A = 0.7821 T = 0.2179	Global
Tegelberg et al. (2021)	rs200392355	6:7452510	beta: 0.16 (0.09–0.22)	*CT*	0.46		1.22E-06	LOC102724234	CT = 0.0005	European (ALFA)
	rs2409703	8:10958526	beta: 0.28 (0.16–0.39)	*C*	0.079		1.61E-06	*XKR6*	T = 0.9066 C = 0.0934	European
	rs11630851	15:76021782	beta: 0.30 (0.18–0.42)	*T*	0.067		9.39E-07	DNM1P35	C = 0.9374 T = 0.0626	European
	rs4444613	20:13340138	beta: -0.28 (-0.38 — -0.18)	*A*	0.087		1.35E-07	TASP1	G = 0.8807 A = 0.1193	European
	rs2003705	20:37763743	beta: -0.16 (-0.23 — -0.10)	*T*	0.2		1.68E-06	LOC107985448	C = 0.7863 T = 0.2137	European
Petty et al., (2023)	rs12036106	1:21998838		T	0.14	C	5.07E-07	RAP1GAP,USP48	C = 0.8604 T = 0.1396	Global
	rs13031512	2:188670717		A	0.11	C	2.60E-06	TFPI,LINC01090	C = 0.9485 A = 0.0515	Global
	rs72870126	4:88859319		G	0.16	A	4.80E-06	MEPE,SPP1	A = 0.7472 G = 0.2528	Global
	rs369717575	4:169522766		TA	0.02	T	1.96E-06	PALLD	(A)8 = 0.9773 delA = 0.0227	Global
	rs36793	5:109328537		T	0.94	C	2.07E-06	LINC01848,TMEM232	C = 0.0946 T = 0.9054	Global
	rs148550758	6:168124558		C	0.02	T	2.04E-06	LINC02487,LINC01558	T = 0.9846 C = 0.0154	Global
	rs7835237	8:23877060		G	0.03	C	3.62E-06	STC1,ADAM28	C = 0.9663 G = 0.0337	Global
	rs12800372	11:68871815		C	0.25	G	4.34E-06	TPCN2,LOC338694	G = 0.8189 C = 0.1811	Global

OR, Odds Ratio; CI, confidence Interval; MAF, minor allele frequency; EAF, effect allele frequency. Note. The included studies not listed in this SNP tables as they do not have SNPs that reach our criteria (5E-06). The highlighted 3 SNPs are from gene SIGLEC5.

### Data synthesis

Table 2 summarized every study and more detailed study characteristics can be viewed in the S6 Table in S2 File. The majority (n = 13) of studies included populations from Europe (German, Finish, Italian, Spanish, Dutch, Finnish, Turkish) and America (European American, American African, Caucasian, non -Hispanic Caucasian, Hispanic and Latino), with seven studies included mixed ethnicity population and six studies included only European or non-Hispanic Caucasian. There were also two studies included samples from East Asia only (Korean and Japanese [33, 37].

Out of these 15 studies, there are six studies focused on the chronic periodontitis, two studies focused on aggressive periodontitis, one study focused on apical periodontitis and one focused on periodontal pocketing. The rest of 5 studies were interested in periodontitis regardless of periodontitis type or including multiple types of periodontitis. The definition and measurement of periodontitis were also varied with most of 15 studies employing full-mouth dental examination performed by trained examiner or dentists and some studies also including radiographs. Eight studies utilised or incorporate different versions of criteria from the Centres for Disease Control and Prevention and American Academy of Periodontology (CDC-AAP) definition [47–51] in which one study measured two sites per tooth [33], two studies measured four sites per tooth [32, 46], two studies measured six sites per tooth [42, 44] and three studies did not specify. Some studies also included radiographs such as x ray. The detailed study characteristic can be viewed in Table 2.

The majority of studies analysed periodontitis as a binary phenotype (case/control), while five studies analysed periodontitis as a continuous variable or included linear regression as part of their analysis to investigate the risk SNPs for periodontitis related traits [32, 38, 44–46]. Two studies had no GWAS significant SNPs found nor the SNPs reaching our lowered suggestive significance threshold for SNP inclusion [43, 46]. Therefore, these two studies were excluded in the next step of SNPs extraction in Table 3. Except for Shungin et al. 2019, no studies have provided a *full list of summary statistic* (total sample size, number of cases, number of controls, odds ratios, 95% confidence interval or standard errors, p-value) but only reported top signal SNPs. It is also noted that some datasets were used in multiple studies (e.g. ARIC data was used in [42, 44, 45].

Table 3 reported all the top signal SNPs extracted from the remaining 13 studies with full details, where 11 risk SNPs (rs242016, rs242014, rs10491972, rs242002, rs2978951, rs2738058, rs4284742, rs729876, rs149133391, rs1537415, rs12461706) were associated with periodontitis at conventional GWAS significance level (*p<5x10*^*-8*^), plus 41 SNPs that reached the suggestive level of significance *(p<5x10*^*-6*^). Although there are no common SNPs reported across study, three large-scale study reported three genome-wide significant SNPs (i.e., rs4284742 effect allele [G], rs11084095 [A], rs12461706 [T]) from the gene SIGLEC5 [34, 39, 45].

## Discussion

This systematic review has identified, critically evaluated, and synthesized genetics evidence from 15 publicly available GWAS studies on periodontitis, published between 2010 and 2023. The majority of studies had good quality, but 10 out of 15 were small studies with a sample size of less than 10,000. A total of 11 SNPs (rs242016, rs242014, rs10491972, rs242002, rs2978951, rs2738058, rs4284742, rs729876, rs149133391, rs1537415, rs12461706) at genome-wide conventional significant level (*p<5x10*^*-8*^) and 41 SNPs at suggestive level (*p<5x10*^*-6*^) were associated with the risk of having periodontitis. In which, three SNPs from three large studies (i.e., rs4284742 [G], rs11084095 [A], rs12461706 [T]) were reported in a same gene—SIGLEC5.

This systematic review has identified several risk variants associated with periodontitis from existing GWAS studies. However, there was huge heterogeneity among study designs and methodologies: sample sizes varied from hundreds to hundred thousand (with the proportion of cases varied from 7.2% to 73.4%, Table 2), ethnicity and population differences, especially, the periodontitis measurements and definitions which may have impact on the GWAS results. The periodontal measurements in the included studies varied from self-reported questionnaire, clinical examination to radiographs, full month examination to half mouth examination, and even studies using same criteria (e.g., CDC-AAP) employed measurements varied from on six sites per tooth to two sites per tooth. Although half mouth examination and fewer sites measurement could lead to more efficient measurement processes, risk of misclassification remains. By utilising questionnaire in measurements, self-reporting bias may also contribute to either underestimation or overestimation of the number of cases. For example, Shimizu et al. 2015 recruited and measured controls separately using self-reported health questionnaire, which may cause underestimation of cases from control participants and may contribute to reduced power to identify genome-wide significant SNPs. Although it is unclear to what extent these heterogeneities in periodontitis definition and measurements would lead to heterogeneity in the GWAS results across studies, future GWAS on periodontitis could benefit from more detailed measurements on periodontal conditions and use of standardised classification criteria. The 2018 periodontal status classification is advocated for use, and proposals have been made to further refine the use of current periodontitis classification to enhance epidemiological data collection and analysis [52]. In addition, more GWAS studies in different ethnic groups, with larger sample sizes and considering covariates are also important for observing the potential for ethnic differences on GWAS results or susceptibility of periodontitis and observe genome-wide significant SNPs.

Heterogeneity in the methods and results reporting was also noted. The current 15 studies incorporated multiple study designs such as inclusion of multiple GWA stages (e.g., 8 studies included discovery and replications). Meanwhile it has been noted that the replication stage had relatively small sample size than discovery stage with five studies included replication stage that have had more than 1,000 participants. However, insufficient sample size in the replication stage could lead to both type I and type II error in the replication results [53]. Meanwhile, it is also important to replicate the results from the combined analysis stage (meta-analysis of discovery and validation stage), which is also missing in the included studies. Future study could utilise better approaches to assess the reproducibility such as the meta-analysis model-based assessment [53]. In terms of results reporting, several included studies did not report the coefficient beta or odds ratio, the standard error or confidence interval, and effect allele of the reported SNPs. This missing information leads to difficulty during data synthesis. Genomic inflation scores were also missing in few studies and not all studies reported or not conducted all quality control steps selected from GWAS quality control guidance. A standardized guideline and consensus on GWAS reporting may be needed to uniform the GWAS report.

To date, there is no guideline in PRISMA on how to perform systematic review on GWAS studies, except Winkler 2014 suggested protocol for genome-wide association meta-analyses in terms of the quality control and conduction of such analyses, requiring the availability of full SNPs and associated statistics reported, allele frequencies, and population stratification [31]. Analytic tools like METAL might estimate the pooled effect for overlapping samples, but it needs genome-wide data to perform appropriate estimation. In addition, a standard and more detailed and widely acceptable quality assessment tool for GWAS is also needed. Although Q-genie tool has been used to assess validity and reliability here, it was designed for genetic association studies, not particularly for GWAS. An assessment tool specifically for GWAS with clear guidance on scoring would be beneficial not only for the assessors but also for the readers to better understand quality assessment criteria.

Form the included 15 studies, three studies based on populations of German, Dutch, European American, Turkish and Asian with sample size > 10,000 participants were commonly discovered three unique SNPs in the gene SIGLEC5, where the effect alleles of all three SNPs has been reported for their protective effect on periodontitis [34, 39, 45]. A recent study has investigated the three genome-wide significant SNPs in the region of SIGLEC5 and shown an impact on SIGLEC5 expression indicating that SIGLEC5 is indeed the target gene for the signal [54]. SIGLEC5 codes for sialic acid-binding Ig-like lectins as a transmembrane inhibitory receptor and is responsible for binding sialic acids and sialic acid-containing glycan ligands. It is expressed in cells in the innate immune system and plays a role in inflammation regulation, both in infection and wound healing [55]. They observed SNP rs11084095 at SIGLEC5 can influence ERG binding and enhancer activity [54], where ERG is important for endothelial homeostasis, including acute response to injury and repair of the endothelium [56]. Meanwhile, the SNP rs12461706 was found in complete linkage disequilibrium with rs11084095. In addition, SNP rs4284742 has been shown to affect MAFB binding affinity, with the common allele enhancing the binding affinity compared to the alternative allele [54]. MAFB is suggested to be associated with the activation of SIGLEC5 expression and contribute to early-onset periodontitis. Further investigation of SIGLEC5 in periodontitis pathologies and intervention targeting the biological pathway underpinned by SIGLEC5 may contributes to both aetiology understanding and disease treatment [57]. In addition to these three SNPs from the same gene region, the rest of the 8 SNPs may also contribute to the periodontitis, however, further GWAS replication with larger sample size may be needed.

The three SNPs in SIGLEC5, two of them were found from both aggressive and chronic periodontitis and one were found from mixed definition defined periodontitis (i.e., including both self-reported and also clinical definition defined periodontitis). This suggested that the SIGLEC5 and the three SNPs may play fundamental roles in all types of periodontitis instead of some specific periodontitis and investigation on SIGLEC 5 may contributes to all type of periodontitis treatment and common pathology understanding. According to the GWAS catalog, most of GWAS significant SNPs found on 15 studies were reported only for periodontitis, except rs2738058 was also found in kidney diseases in Chinese population (i.e., IGA glomerulonephritis) [58]. Meanwhile, several mapped genes of these GWAS significant SNPs were also found significant in IGA glomerulonephritis (e.g., DEFA9P and DEFA10P [58]), neuropsychological conditions (e.g., TMF1P1 [59]), despite of reporting uncommon SNP. These may suggest somewhat genetic similarity between periodontitis and these conditions but further investigation on their relationship with periodontitis still needed.

In comparison to the previous systematic review on the heritability of periodontitis [19], our focus lies more heavily on the methodology utilised in GWAS and synthesis of results. Moreover, comparing with the review article of genetics of periodontitis by Shaddox et al. 2021, we employed a systematic approach and included a greater number of studies than the prior review. It is noted that 6 out of 8 studies that used chronic periodontitis as disease phenotype did not meet the required sample size of >10K cases for such disease with low heritability, except the studies Munz et al. 2017 and Munz et al. 2019. For the 8 studies that used aggressive periodontitis with high heritability as disease definition, smaller sample size is usually acceptable but no studies showed any common risk variants, indicating a potential of false positive results.

The findings that NO common SNPs were consistently reported through all the included studies highlighted a significant level of heterogeneity in the results obtained from GWAS of periodontitis. Given this lack of repeatability in GWAS finding, any identified genetic variants must be interpretated with caution. Furthermore, we observed a reluctance within the dental research community to share GWAS results. Only one study (Shungin et al. 2019) provided a comprehensive list of SNPs statistics from their GWAS of periodontitis, while the remaining studies offered only a limited number of top-signaled SNPs, with some providing incomplete statistics such as lacking odds ratios or 95% confidence intervals. When we attempted to obtain full SNP statistics from these studies, they either declined or did not respond, underscoring a significant transparency issue in current dental research practices. In many common diseases within the medical field, guidelines exist mandating GWAS data sharing as a standard practice expected by funders and publishers. We call for similar guidelines to be established in dental research, requiring the sharing of GWAS statistics from published work to facilitate advancement within the field.

The current study has several strengths, such as summarizing existing risk variants in the literature and discussed the study design of current GWAS on periodontitis, and presenting each study clearly with its database used, population, and GWAS testing method used. However, there are also some limitations to consider. Publication bias should be kept in mind while interpreting the results, as only publicly available GWAS studies were included in the review [26]. Additionally, potential biases from the selected studies may exist, such as those from the periodontitis definition, and variation of methodology applied (e.g. number of covariates adjusted in the association analysis). The current study failed to obtain access to full GWAS summary statistics to perform meta-analysis which could contribute to resolve small sample size in many studies and detect risk variants in combined sample. For example, SNPs with consistently border-line association with periodontitis could have been missed, which may have in theory become statistically significant in meta-analysis if most studies had provided a full list of statistics for all SNPs. In addition, since the majority of included studies sampled were from white population (European, European American, Caucasian etc.) and only few of them included Latino, Black, Asian or mixed ethnicity, it is difficult to draw conclusion based on each ethnicity group. More studies investigating Black and Asian populations could help to understand the underlying ethnicity difference of periodontitis, and future GWAS should also provide summary statistics in repository such as GWAS catalog to facilitate further meta-analysis.

## Conclusions

To conclude, our systematic review of 15 GWAS studies on periodontitis identified 11 SNPs were at genome-wide significance *p<5x10*^*-8*^ level. Variants near or in the gene region SIGLEC5 were reported most frequently (i.e. in three large scale studies) for its potential role on periodontitis. These results imply potential therapeutic targets pathway underlined by the SIGLEC5. Further investigation on this gene could contributed to the periodontitis treatment approach design. However, the heterogeneity on study design, study sample size and target population between studies has been noted. To improve our understanding of periodontitis and support the development of effective treatment options, more high-quality and homogeneous methodology used in GWAS studies are needed. These studies should use standardized periodontitis definitions and assessment tools, have larger sample sizes, and include different ethnicities. Data repository of GWAS results should be made available so that further meta-analysis can be possible, especially in dental research, to ensure research transparency and reproducibility. These efforts will contribute to greater understanding of this oral disease and ultimately benefit public health.

## Supporting information

S1 Checklist PRISMA checklist, S8 Table. (DOCX)

S1 File S1-S4, S7 Tables. (DOCX)

S2 File S5 and S6 Tables. (XLSX)
