# Supplementary material for: Genome-wide association studies on periodontitis: A systematic review
Source: PLoS One. 2024 Sep 6;19(9):e0306983. doi: 10.1371/journal.pone.0306983 (PMC11379206; doi:10.1371/journal.pone.0306983)
Supplement: S1 File — (DOCX) [file pone.0306983.s002.docx]

**Supplement:** Systematic review of genome-wide association studies of periodontitis

**Table S1. The search strategies and results summary in the GWAS catalog**

| Search terms & search strategy | Search results |
| --- | --- |
| Periodontal disease | 3 |
| periodontitis | 13 |
| periodon | 0 |
|  |  |

**Table S2. The search strategies and results summary in PubMed**

| category | Search strategy | results |
| --- | --- | --- |
| 1. periodontitis | ((periodontal disease[Title/Abstract]) OR (periodontitis[Title/Abstract])) OR (periodon*[Title/Abstract]) |  |
| 2. GWAS | ((((((GWA[Title/Abstract]) OR (genome wide[Title/Abstract])) OR (GWAS[Title/Abstract])) OR (genome wide association[Title/Abstract])) OR (whole genome association[Title/Abstract])) OR (WGA[Title/Abstract])) OR (WGAS[Title/Abstract]) |  |
| 1 and 2 | (((periodontal disease[Title/Abstract]) OR (periodontitis[Title/Abstract])) OR (periodon*[Title/Abstract])) AND (((((((GWA[Title/Abstract]) OR (genome wide[Title/Abstract])) OR (GWAS[Title/Abstract])) OR (genome wide association[Title/Abstract])) OR (whole genome association[Title/Abstract])) OR (WGA[Title/Abstract])) OR (WGAS[Title/Abstract])) | 202 |

**Table S3. The search strategies and results summary in ScienceDirect**

| Search terms/search strategies | search field | No. of Search Results |
| --- | --- | --- |
| WGA periodontal disease | Title, abstract or author-specified keywords | 1 |
| WGA periodontitis |  | 0 |
| whole genome association periodontal disease |  | 1 |
| whole genome association periodontitis |  | 0 |
| genome wide association periodontal disease |  | 5 |
| genome wide association periodontitis |  | 10 |
| GWAS periodontitis |  | 6 |
| GWAS periodontal disease |  | 4 |
| GWA periodontal disease |  | 4 |
| GWA periodontitis |  | 6 |
| WGAS periodontitis |  | 0 |
| WGAS periodontal disease |  | 1 |

**Table S4.. The search strategies and results summary in EMBASE, GLOBAL HEALTH, MEDLINE (via OVID)**

|  | search terms | results |
| --- | --- | --- |
| 1 | genome-wide association.ab. or genome-wide association.ti. or genome-wide association.kf. | 102642 |
| 2 | periodontitis.ab. or periodontitis.ti. or periodontitis.kf. | 75165 |
| 3 | periodontal disease.ab. or periodontal disease.ti. or periodontal disease.kf. | 44806 |
| 4 | periodon*.ab. or periodon*.ti. or periodon*.kf. | 176245 |
| 5 | GWA.ab. or GWA.ti. or GWA.kf. | 3544 |
| 6 | genome wide.ab. or genome wide.ti. or genome wide.kf. | 264521 |
| 7 | whole genome association.ab. or whole genome association.ti. or whole genome association.kf. | 1272 |
| 8 | WGA.ab. or WGA.ti. or WGA.kf. | 10212 |
| 9 | WGAS.ab. or WGAS.ti. or WGAS.kf. | 132 |
| 10 | GWAS.ab. or GWAS.ti. or GWAS.kf. | 60911 |
| 11 | 2 or 3 or 4 | 176245 |
| 12 | 1 or 5 or 6 or 7 or 8 or 9 or 10 | 287085 |
| 13 | 11 and 12 | 491 |

**Table S5. Summary of the quality control steps taken in included studies**

(please see the excel file)

**Table S6. Summary of study characteristics**

(Please see the excel file )

**Table S7. PRISMA 2020 abstract checklist**

| **Section and Topic** | **Item #** | **Checklist item** | **Reported (Yes/No)** |
| --- | --- | --- | --- |
| **TITLE** | | |  |
| Title | 1 | Identify the report as a systematic review. | Yes |
| **BACKGROUND** | | |  |
| Objectives | 2 | Provide an explicit statement of the main objective(s) or question(s) the review addresses. | Yes |
| **METHODS** | | |  |
| Eligibility criteria | 3 | Specify the inclusion and exclusion criteria for the review. | Yes |
| Information sources | 4 | Specify the information sources (e.g. databases, registers) used to identify studies and the date when each was last searched. | Yes |
| Risk of bias | 5 | Specify the methods used to assess risk of bias in the included studies. | Yes |
| Synthesis of results | 6 | Specify the methods used to present and synthesise results. | Yes |
| **RESULTS** | | |  |
| Included studies | 7 | Give the total number of included studies and participants and summarise relevant characteristics of studies. | Yes |
| Synthesis of results | 8 | Present results for main outcomes, preferably indicating the number of included studies and participants for each. If meta-analysis was done, report the summary estimate and confidence/credible interval. If comparing groups, indicate the direction of the effect (i.e. which group is favoured). | Yes |
| **DISCUSSION** | | |  |
| Limitations of evidence | 9 | Provide a brief summary of the limitations of the evidence included in the review (e.g. study risk of bias, inconsistency and imprecision). | Yes |
| Interpretation | 10 | Provide a general interpretation of the results and important implications. | Yes |
| **OTHER** | | |  |
| Funding | 11 | Specify the primary source of funding for the review. | No (mentioned in acknowledgement section not abstract) |
| Registration | 12 | Provide the register name and registration number. | No (Mentioned in method section not abstract) |

*From:*  Page MJ, McKenzie JE, Bossuyt PM, Boutron I, Hoffmann TC, Mulrow CD, et al. The PRISMA 2020 statement: an updated guideline for reporting systematic reviews. BMJ 2021;372:n71. doi: 10.1136/bmj.n71
